# Development of a pediatric formulation for treatment of *P. falciparum* malaria: Coartem^®^ (artemether-lumefantrine) Dispersible

**DOI:** 10.1186/1475-2875-13-S1-P7

**Published:** 2014-09-22

**Authors:** Quique Bassat, Bernhards Ogutu, Abdoulaye DJimde, Kirstin Stricker, Kamal Hamed

**Affiliations:** 1 Barcelona Centre for International Health Research (CRESIB), Hospital Clínic, Universitat de Barcelona, Barcelona, Spain; 2 Centre for Clinical Research, Kenya Medical Research Institute, Nairobi, Kenya; 3 Malaria Research and Training Center, University of Science, Techniques and Technologies of Bamako, Bamako, Mali; 4 Novartis Pharma AG, Basel, Switzerland; 5 Novartis Pharmaceuticals Corporation, East Hanover, NJ, USA

## Background

Pediatric artemisinin-based combination therapy formulations have the potential to improve the effectiveness and accuracy of dosing in young children. Coartem^®^ (artemether-lumefantrine; AL) Dispersible was developed and launched in partnership with the Medicines for Malaria Venture for the treatment of uncomplicated *Plasmodium falciparum* malaria and is the first pediatric antimalarial to receive Swissmedic approval and meet WHO specifications for use in infants and children ≥5 kg.

## Materials and methods

PubMed, Ovid and clinical trial registry databases were explored for publications on AL dispersible formulation in children with acute uncomplicated *P. falciparum* malaria, and 11 publications (6 original and 5 review articles) involving 674 infants and children were identified.

## Results

In a palatability study, sweet tasting cherry was the preferred flavor by children for AL dispersible. Pharmacokinetic profile of AL dispersible was comparable to AL crushed tablets. Efficacy and safety of dispersible formulation versus crushed tablet was evaluated in a large, randomized, multicenter study in 5 sub-Saharan African countries. Efficacy and acceptability of AL dispersible were also compared to dihydroartemisinin-piperaquine (DP) pediatric in another open-label, randomized study in Kenya. A total of 674 children were randomized in both studies to receive AL dispersible with a mean age of 38.5 months, body temperature of 38.1°C and parasite density 41,974/μl. PCR-corrected cure rate was 97.8% at day 28 and 96.4% at day 42; similar to AL crushed tablets and DP in respective studies. PCR-corrected cure rate at day 28 was not related to food intake, although consumption of milk/low fat meal increased lumefantrine bioavailability compared to no food. Efficacy of AL dispersible was comparable in children with different body weights. Median parasite and fever clearance times were 34.3 and 7.9 hours, respectively. The safety profile of AL dispersible was similar to that of crushed tablets. Acceptability of AL dispersible was significantly better than DP pediatric (ease of use: *P* = 0.007; taste of medicine: *P* = 0.001).

## Conclusions

AL dispersible was specifically tailored for the pediatric population and offers a convenient formulation with efficacy and safety similar to that of standard crushed AL tablets.

